# Childhood trauma and bullying-victimization as an explanation for differences in mental disorders by sexual orientation

**DOI:** 10.1016/j.jpsychires.2021.02.046

**Published:** 2021-02-22

**Authors:** Laura Baams, Margreet ten Have, Ron de Graaf, Peter de Jonge

**Affiliations:** a University of Groningen, the Netherlands; b Netherlands Institute of Mental Health and Addiction (Trimbos Institute), the Netherlands

**Keywords:** Sexual minority, Childhood trauma, Bullying, Mental disorders

## Abstract

Sexual minority individuals are more likely to have mental disorders, including mood, anxiety, and substance use disorders, compared to heterosexual individuals. Whether experiencing trauma or bullying-victimization during childhood explains these differences is currently unclear. We used a psychiatric epidemiological general population-based study to assess whether childhood trauma severity and bullying-victimization before age 16 explains the difference by sexual attraction in mental disorders. Data from the Netherlands Mental Health Survey and Incidence Study-2 (NEMESIS-2; N = 6392) were used to examine (1) whether same/both-sex attraction and predominantly other-sex attraction is linked to self-reports of childhood trauma (types and severity) and bullying-victimization, and (2) whether these experiences explain differences between these groups in lifetime and 12-month prevalence of DSM-IV disorders assessed by the Composite International Diagnostic Interview 3.0. Same/both-sex attracted individuals reported a higher childhood trauma severity score compared to exclusively other-sex attracted individuals (*B =* 0.93, *SE* = 0.20, p < .001), and were more likely to report bullying-victimization (*OR* = 2.51 95%CI[1.68, 3.74]). DSM-IV disorders were more prevalent among same/both-sex attracted individuals than among exclusively other-sex attracted individuals (ORs ranged from 1.57 to 4.68). There were no differences in DSM-IV disorders for predominantly other-sex attracted individuals. Childhood trauma severity explained between 9.0% and 57.0% of significant indirect associations between same/both-sex attraction and DSM-IV disorders. Sexual minority individuals experience more types of, and more severe childhood trauma, and are more likely to experience bullying-victimization. These negative experiences partly explained disparities in mental disorders.

## 1 Introduction

Despite increasing social acceptance of sexual diversity and same-sex relationships in Western countries (Kuyper et al., 2013), disparities in mental health between sexual minority and heterosexual individuals remain (Lucassen et al., 2017). The high prevalence of mood, anxiety, and substance use disorders among sexual minority individuals (Bostwick et al., 2010; Lucassen et al., 2017; McCabe et al., 2009) is thought to be explained by experiences of childhood trauma and bullying-victimization (Baams, 2018; Martin-Storey and Fish, 2019; Zou and Andersen, 2015).

### 1.1 Sexual minority status and mental disorders

Sexual minority individuals show a higher prevalence of common mental disorders, such as mood, anxiety, and substance use disorders. For example, in both the first and second Netherlands Mental Health Survey and Incidence Study (NEMESIS and NEMESIS-2), mental disorders were more prevalent in adults who reported same- or both-sex attractions or sexual behavior with same-sex partners compared to adults who reported exclusively other-sex attractions or behaviors (Sandfort et al, 2001, 2014). Further, in a US-representative sample of adults, sexual minority individuals were more likely to have lifetime or 12-month mood or anxiety disorders, and more likely to have 12-month substance use disorders (Bostwick et al., 2010; McCabe et al., 2009).

Although research on sexual minority individuals is often limited to including people who experience attraction to people of the same sex, or to both men and women, there is increasing attention for individuals who report a "mostly heterosexual" orientation or experience predominantly other-sex attraction (Vrangalova and Savin-Williams, 2012). Research comparing mostly heterosexual individuals with heterosexual individuals shows that mostly heterosexual men and women report higher levels of depressive symptoms (Krueger et al., 2018), and that mostly heterosexual women are more likely to report substance use, including substance dependence (Hughes et al., 2015). Although research accounting for mostly heterosexual identities is still limited, there is indication that this group has distinct experiences with both mental health (Vrangalova and Savin-Williams, 2012) and victimization (Austin et al., 2008; Zou and Andersen, 2015) and should not be conflated with an exclusively heterosexual identity.

### 1.2 Childhood trauma and bullying-victimization

Childhood trauma and bullying-victimization are important contributors to psychopathology in childhood and adulthood (Hughes et al., 2017; Kalmakis and Chandler, 2015; Takizawa et al., 2014; ten Have et al., 2019). For example, a meta-analytic study showed that individuals who experienced childhood maltreatment had two times higher odds of experiencing major depressive disorder, and 2.7 times higher odds of experiencing an anxiety disorder in adulthood (Li et al., 2016). In addition, studies show increased substance use disorders in (young) adults who experienced childhood maltreatment compared to those who did not (Afifi et al., 2012; Hughes et al., 2010; Kendler et al., 2000), even after adjusting for (socio)demographic factors such as income and educational attainment or lifetime mood and anxiety disorders (Afifi et al., 2012), and general family functioning (Kendler et al., 2000). Further, bullying-victimization during childhood is linked to internalizing and externalizing mental health problems in adulthood, including depressive symptoms (Sigurdson et al., 2015).

Common definitions of trauma state that events should be actual or potentially life-threatening to be considered traumatic. However, scholars have pointed to the problems associated with using such a strict definition to decide what experiences might be considered traumatic (Pantalone et al., 2020). This narrow definition may be especially problematic for sexual minority individuals whose experiences may not meet the criteria for trauma events (e.g., discrimination, rejection, and bullying), but are clearly linked to mental disorders (Gevonden et al., 2014; Islam et al., 2020; Moore et al., 2017; Pantalone et al., 2020; Takizawa et al., 2014), including posttraumatic stress disorder (Bandermann and Szymanski, 2014). Researchers have suggested that it may be the persistent nature of these experiences that accumulates to trauma (Pantalone et al., 2020).

Theoretical (McLaughlin et al., 2012; Meyer, 2003) and empirical work (Bostwick et al., 2014; Hatzenbuehler et al., 2010) suggests that sexual orientation disparities in mental disorders are partly explained by increased rates of victimization and discrimination among sexual minority individuals. For example, a meta-analytic study showed that sexual minority adolescents had almost three times higher odds of reporting childhood sexual abuse, 2.3 times higher odds of reporting physical abuse, and 2.7 times higher odds of reporting peer victimization; emotional abuse was not assessed (Friedman et al., 2011). In addition, in a representative study among adolescents (McLaughlin et al., 2012) and a convenience sample of adults (Andersen et al., 2015) abuse and bullying-victimization explained differences in physical and mental health between heterosexual and sexual minority individuals.

### 1.3 The current study

To understand whether experiences with childhood trauma and bullying-victimization before age 16 explain the difference between same/both- and exclusively other-sex attracted, and predominantly other- and exclusively other-sex attracted individuals in mental disorders, we used data from a psychiatric epidemiological general population-based study. We assessed differences in lifetime and 12-month prevalence of DSM-IV disorders for exclusively other-sex attracted versus predominantly other-sex attracted and same/both-sex attracted individuals. Second, we assessed whether childhood trauma severity and bullying-victimization explained these differences. To our knowledge, this is the first study to assess whether these childhood experiences explain differences by sexual orientation in a sample of adults from the Netherlands. We hypothesize that compared to exclusively other-sex attracted individuals, same/both-sex attracted individuals will be more likely to have experienced childhood trauma and bullying-victimization, and that childhood trauma severity and bullying-victimization will explain differences in the lifetime and 12-month prevalence of DSM-IV disorders. Based on the limited literature on mostly heterosexual individuals' risks of mental disorders and childhood trauma, we have no hypotheses about the differences between predominantly other- and exclusively other-sex attracted individuals.

## 2 Method

### 2.1 Participants

NEMESIS-2 is a prospective study in a Dutch-speaking sample aged 18–68 years old, drawn from the general Dutch population (de Graaf et al., 2010). A multistage, stratified random sampling procedure was applied. First, a random sample of municipalities and then a random sample of addresses from these municipalities were drawn (addresses of institutions were excluded). Based on the most recent birthday within the household, a random adult aged 18-68 years old who spoke the Dutch language was selected for participation. The authors assert that all procedures contributing to this work comply with the ethical standards of the relevant national and institutional committees on human experimentation and with the Helsinki Declaration of 1975, as revised in 2008. All adult participants provided written informed consent to participate in this study.

The total sample comprised *N* = 6646 participants who completed the baseline wave of NEMESIS-2 between November 2007 and July 2009, with a response rate of 65.1%. A total of *N =* 6393 participants completed the question about sexual attraction and were included in the current study. This sample had a mean age of 44.2 *(SD* = 12.5) and 55.1% identified as women, while 44.9% identified as men. The majority of the sample attended higher professional education or university (35.8%), and others attended higher secondary education (32.3%), lower secondary education (27.3%), or primary education (4.6%). A small group of participants (5.7%) were of non-western origin, defined as respondent or at least one parent not born in Western Europe or North America (mainly of Surinamese, Antillean, Turkish and Moroccan origin). 91.0% of participants reported being exclusively attracted to the other sex, 6.5% reported being predominantly attracted to the other sex, 1.7% reported being attracted exclusively or predominantly to the same sex, and 0.8% reported being attracted to men and women equally (See Table 1).

### 2.2 Measures

#### DSM-IV disorders

The Composite International Diagnostic Interview (CIDI) version 3.0 was used to assess lifetime and 12-month occurrence of DSM-IV disorders. Disorders included in the current study are major depression, dysthymia, bipolar disorder, panic disorder, agoraphobia (without panic disorder), social phobia, specific phobia, generalized anxiety disorder, substance abuse and dependence (combining alcohol and drug use). In the current study we also included the disorder categories: any mood disorder, any anxiety disorder, and any substance use disorder, and we assessed the occurrence of any Axis-1 disorder and total number of Axis-1 disorders (as mentioned above, including substance use disorders). Previous studies found that the CIDI 3.0 assesses these mental disorders with good validity in comparison to blinded clinical reappraisal interviews (Haro et al., 2006). See de Graaf et al. (2010) for an extended description of the CIDI 3.0. See Supplemental Table 1 for the prevalence of DSM-IV disorders by sexual attraction reports.

#### Childhood trauma

Childhood trauma was assessed with a questionnaire developed for NEMESIS (ten Have et al., 2019). The questionnaire asked about the occurrence of emotional abuse (ignored or unsupported), psychological abuse (yelled at, insulted or threatened), physical abuse (kicked, hit, bitten or hurt), and sexual abuse (any unwanted sexual experience) before the age of 16. If participants indicated that any of these forms of trauma had occurred, they were asked how often it had occurred, on a scale of 1 (once) to 5 (very often). These frequencies were categorized as absent (0), once or sometimes (1), regularly, often, and very often (2). By combining the occurrence of trauma (0–4) and frequency of trauma (0–2), we created a childhood trauma severity score, ranging from 0 to 8, with a higher score indicating more severe childhood trauma. See ten Have et al. (2019) for an extended description of this measure. Table 2 presents the occurrence of childhood trauma by sexual attraction.

#### Bullying-victimization

Bullying-victimization was assessed with one item "Before turning 16 years old, were you bullied on a regular basis?", with answer options yes (1) and no (0). Table 2 presents the occurrence of bullying-victimization by sexual attraction.

#### Sexual orientation

Sexual orientation was assessed with one item about sexual attraction "Are you sexually attracted to men, women, or both?" with response options Exclusively to women, Predominantly to women, Women and men equally, Predominantly to men, Exclusively to men. For the purpose of this study, same-sex attraction refers to participants who reported exclusively or predominantly being attracted to people of the same sex, both-sex attraction refers to participants who reported equal attraction to women and men, predominantly other-sex attraction refers to participants who reported being attracted to predominantly another sex, and exclusively other-sex attracted refers to participants who reported exclusively being attracted to people of another sex. Due to small cell sizes, same- and both-sex attracted participants were combined into a same/both-sex attracted group for the purpose of the current analyses.

### 2.3 Statistical analyses

To assess differences by sexual attraction in the occurrence and severity of childhood trauma, we conducted (logistic) regression analyses. To assess differences in the occurrence of DSM-IV disorders we conducted logistic regression analyses for lifetime and 12-month occurrence. To examine whether childhood trauma severity and bullying-victimization explained the difference in the occurrence of DSM-IV disorders, we added childhood trauma severity and bullying-victimization, separately, to the logistic regression analyses if the initial difference by sexual attraction was significant. Next, we assessed indirect effects of childhood trauma severity and bullying-victimization using the SEM command in Stata, and calculated the percentage explained (Rijnhart et al., 2019). All analyses controlled for age, gender, and education level. All analyses were conducted in Stata version 16 and accounted for the complex survey design, including weights, strata (region), and primary sampling units (municipalities).

## 3 Results

Results from logistic regression analyses showed that same/both-sex attracted individuals are more likely to have experienced all types of childhood trauma and bullying-victimization, compared to exclusively other-sex attracted individuals. In addition, same/both-sex attracted individuals had a higher childhood trauma severity score compared to exclusively other-sex attracted individuals. There were no significant differences in childhood trauma severity and the likelihood of childhood trauma types and bullying-victimization between predominantly other-sex attracted and exclusively other-sex attracted individuals (see Table 2).

Overall, differences by sexual orientation in DSM-IV disorders showed that same/both-sex attracted individuals were more likely to have lifetime (see Table 3a) and 12-month prevalence (see Table 3b) of any mood disorder, major depression, bipolar disorder (only lifetime), dysthymia (only lifetime), any anxiety disorder, social phobia, panic disorder (only 12-month) any substance use disorder (only lifetime), substance abuse (only lifetime), substance dependence (only 12-month), and any Axis-1 disorder, and *more* Axis-1 disorders compared to exclusively other-sex attracted individuals. There were no significant differences in the likelihood of DSM-IV disorders between predominantly other-sex attracted and exclusively other-sex attracted individuals (see Supplemental Table 2).

Childhood trauma severity and bullying-victimization were added to the models of DSM-IV disorders for which there was a significant difference between same/both- and exclusively other-sex attracted individuals (see Tables 3ab).

These results showed that indirect associations through childhood trauma severity were significant for lifetime any mood disorder (*B* = 0.06, *SE* = 0.01, *p* < .001), major depression (*B* = 0.05, *SE* = 0.01, *p* < .001), bipolar disorder (*B* = 0.01, *SE* = 0.00, *p* = .019), dysthymia (*B* = 0.01, *SE* = 0.00, *p* = .001), any anxiety disorder (*B* = 0.05, *SE* = 0.01, *p* < .001), social phobia (*B* = 0.03, *SE* = 0.01, *p* < .001), any substance use disorder (*B* = 0.03, *SE* = 0.01, *p* < .001), substance abuse (*B* = 0.02, *SE* = 0.01, *p* = .001), any Axis-1 disorder (*B* = 0.07, *SE* = 0.02, *p* < .001), and number of Axis-1 disorders (*B* = 0.19, *SE* = 0.04, *p* < .001). Indirect relations were also significant for 12-month any mood disorder (*B* = 0.03, *SE* = 0.01, *p* < .001), major depression (*B* = 0.02, *SE* = 0.01, *p* < .001), any anxiety disorder (*B* = 0.03, *SE* = 0.01, *p* < .001), social phobia (*B* = 0.01, *SE* = 0.00, *p* < .001), panic disorder (*B* = 0.01, *SE* = 0.01, *p* = .007), substance dependence (*B* = 0.00, *SE* = 0.00, *p* = .028), any Axis-1 disorder (*B* = 0.05, *SE* = 0.01, *p* < .001), and number of Axis-1 disorders (*B* = 0.09, *SE* = 0.02, *p* < .001). For all significant indirect associations, we calculated what percentage of indirect associations could be explained by childhood trauma severity: between 9.0% and 57.0% of indirect associations was explained by childhood trauma severity (see Tables 3ab).

Table 4 shows indirect associations through bullying-victimization for DSM-IV disorders that showed differences by sexual attraction. Indirect associations through bullying-victimization were significant for lifetime any mood disorder (*B* = 0.03, *SE* = 0.01, *p* = .001), major depression (*B* = 0.02, *SE* = 0.01, *p* = .002), bipolar disorder (*B* = 0.01, *SE* = 0.00, *p* = .013), dysthymia (*B* = 0.00, *SE* = 0.00, *p* = .019), any anxiety disorder (*B* = 0.03, *SE* = 0.01, *p* < .001), social phobia (*B* = 0.02, *SE* = 0.01, *p* = .001), any substance use disorder (*B* = 0.01, *SE* = 0.00, *p* = .045), any Axis-1 disorder (*B* = 0.03, *SE* = 0.01, *p* = .001), and number of Axis-1 disorders (*B* = 0.09, *SE* = 0.03, *p* < .001). Indirect relations were also significant for 12-month any mood disorder (*B* = 0.01, *SE* = 0.00, *p* = .004), major depression (*B* = 0.01, *SE* = 0.00, *p* = .025), any anxiety disorder (*B* = 0.02, *SE* = 0.01, *p* < .001), social phobia (*B* = 0.01, *SE* = 0.00, p = .001), any Axis-1 disorder (*B* = 0.02, *SE* = 0.01, *p* = .001), and number of Axis-1 disorders (*B* = 0.04, *SE* = 0.01, *p* = .001). For all significant indirect associations, we calculated what percentage of indirect associations could be explained by bullying-victimization: between 6.6% and 26.8% of indirect associations was explained by bullying-victimization (see Table 4).

## 4 Discussion

Using a psychiatric epidemiological general population-based study, we assessed whether experienced childhood trauma severity and bullying-victimization explained sexual orientation-based differences in the prevalence of mental disorders in the Netherlands. We found that same/both-sex attracted individuals were more likely to have experienced all types of childhood trauma and bullying-victimization, and more severe childhood trauma compared to exclusively other-sex attracted individuals, and that childhood trauma severity and bullying-victimization partly explained differences in mental disorders for same/both-sex attracted individuals. We found no differences in the likelihood of DSM-IV disorders, childhood trauma, and bullying-victimization between predominantly and exclusively other-sex attracted individuals.

The current findings support our hypotheses and correspond with previous research suggesting an important role of childhood trauma and bullying-victimization in the development of mood, anxiety, and substance use disorders in sexual minority individuals (Bostwick et al., 2014; Hatzenbuehler et al., 2010; McLaughlin et al., 2012; Meyer, 2003). Furthermore, our findings contribute to a discussion about definitions of potentially traumatic events for marginalized groups (Pantalone et al., 2020). We assessed childhood maltreatment and bullying-victimization, separately. Although research shows that bullying-victimization is an important factor in the development of mental disorders (Moore et al., 2017; Takizawa et al., 2014), future research should further examine whether the accumulation of such experiences among sexual minority individuals have the same impact on mental health as less frequent perceived or actual life-threatening events.

Further, although we included various traumatic experiences that children and adolescents might have, ranging from bullying-victimization to physical and sexual abuse, there may be other experiences that better explain mental disorders in sexual minority individuals, such as negative life events after age 16, and discrimination (Baams et al., 2015). However, the current study does indicate that sexual minority people experience more childhood trauma and are more likely to experience bullying-victimization and that these experiences explain mental health overall. Further, childhood trauma is thought to be a particularly strong predictor of substance use. Sexual minority individuals may use substances as a way to cope with negative emotions elicited by early traumatic experiences (Fish and Hughes, 2018). In addition, because substance use in the childhood home is an important adverse experience for sexual minority adolescents (Baams, 2018), future research should consider its relevance for the development of substance use disorders and other mental disorders among sexual minority adults.

In contrast to previous research (Austin et al., 2008; Hughes et al., 2015; Krueger et al., 2018; Zou and Andersen, 2015), our study did not show any differences in childhood trauma, bullying-victimization, or mental disorders for individuals who reported being predominantly attracted to the other sex. To our knowledge, only one study in the Netherlands has previously examined experiences of mostly heterosexual individuals (only in comparison to lesbian/gay individuals, Kuyper and Bos, 2016). Future research might examine whether the meaning of "mostly heterosexual" identities differs across contexts and what would be the best way to assess this sexual orientation in a general population of adults.

### 4.1 Strengths and limitations

To our knowledge, this study is the first to assess whether a comprehensive measure of childhood trauma severity explains DSM-IV disorders in a representative sample of adults. In addition, this study also assessed the occurrence of bullying-victimization during childhood, a known predictor of adult mental health (Takizawa et al., 2014). Despite the Netherlands being relatively accepting of sexual diversity (Kuyper et al., 2013), the current study shows clear sexual orientation disparities in childhood trauma and bullying-victimization and their role in mental disorders. The current study also has several limitations to consider. First, respondents in this study were asked about childhood trauma and bullying-victimization retrospectively as is common in this type of research. Although the assessment of these experiences in adulthood is reliable, and mostly leads to false negatives as opposed to false positives (Hardt and Rutter, 2014), respondents may differ in their ability and willingness to report such experiences. In addition, because the current data are cross-sectional and correlational, we cannot infer causality or developmental patterns, or mediation by childhood trauma or bullying-victimization. Second, although all childhood trauma and bullying-victimization questions referred to experiences before the age of 16, we cannot rule out that these events took place after the emergence of mental disorders. Further, additional traumatic experiences may have occurred after the age of 16, and impacted the emergence of mental disorders. Third, with the current study we cannot conclude whether the experiences with abuse and bullying were targeted at someone's sexual orientation or whether respondents were aware of their sexual orientation during this time. However, sexual orientation disparities in victimization emerge already around age 9 (Martin-Storey and Fish, 2019), before many youth become aware of their sexual orientation. Further, focusing on a universal social determinant of health (i.e., childhood trauma) to explain disparities in mental health is an important strength of the current study.

Future research might combine measures of discrimination, bullying-victimization, and childhood trauma to provide a comprehensive view of sexual minority youth experiences. Last, in line with previous research, there were differences in the prevalence of same- and both-sex attraction between men and women. Among sexual minority women, both-sex attraction was most common, while sexual minority men were most likely to report being exclusively attracted to men. Predominantly other-sex attraction was also more common among women than among men. Although research points to bisexual individuals as particularly vulnerable to the experience of childhood trauma (Baams, 2018), and our study also shows patterns of vulnerability for both-sex attracted individuals, we did not have sufficient statistical power to assess disparities for both-sex attracted and same-sex attracted individuals separately.

## 5 Conclusion

Ultimately, this study underlines the increased risk of experiencing childhood trauma and bullying-victimization for sexual minority individuals, and its impact on mental health later in life. The findings of the current study add to a growing body of literature explaining some of the mental health disparities that we observe in sexual minority adults, and helps us better understand the urgency of intervening before these mental disorders develop.

## Supplementary Material

Supplementary data to this article can be found online at https://doi.org/10.1016/j.jpsychires.2021.02.046.

Appendix A

## Figures and Tables

**Table 1:** Reports of sexual attraction for men and women.

	Total	Men	Women
Sexual attraction:	*n* (%)	*n* (%)	*n* (%)
Exclusively other-sex attracted	5818 (91.00)	2682 (93.45)	3136 (89.02)
Predominantly other-sex attracted	413 (6.46)	115 (4.01)	298 (8.46)
Both-sex attracted	53 (0.83)	11 (0.38)	42 (1.19)
Predominantly same-sex attracted	31 (0.48)	12 (0.42)	19 (0.54)
Exclusively same-sex attracted	78 (1.22)	50 (1.74)	28 (0.79)

*Note.* For the purpose of our focal analyses, both-sex attracted, predominantly same-sex attracted, and exclusively same-sex attracted individuals were combined into a same/both-sex attracted group.

**Table 2:** Types and severity of childhood trauma and bullying-victimization by sexual attraction and socio-demographic variables (weighted).

	Emotional abuse (*N* = 6363)	Psychological abuse (*N* = 6374)	Physical abuse (*N* = 6383)	Sexual abuse (*N* = 6379)	Childhood trauma severity^a^ (*N* = 6392)	Bullying-victimization (*N* = 6388)
	%	%	%	%	*M* (*SE*)	%
Exclusively other-sex attracted	13.97	16.98	9.01	7.22	0.45 (0.01)	14.94
Predominantly other-sex attracted	17.32	19.31	9.46	10.64	0.55 (0.06)	19.97
Both-sex attracted	29.12	21.02	12.47	23.21	0.90 (0.25)	23.49
Predominantly same-sex attracted	43.73	27.25	11.72	28.56	1.09 (0.18)	15.88
Exclusively same-sex attracted	33.01	29.24	25.55	10.03	0.91 (0.09)	42.06
	aOR [95%CI]	aOR [95%CI]	aOR [95%CI]	aOR [95%CI]	*B* (*SE*)	aOR [95%CI]
Sexual attraction (ref: exclusively other-sex attracted)						
Predominantly other-sex attracted	1.14 [0.85, 1.53]	1.11 [0.83, 1.49]	1.02 [0.68, 1.52]	1.21 [0.82, 1.77]	0.14 (0.11)	1.30 [0.94, 1.82]
Same/both-sex attraction	**3.08 [2.00, 4.73]**	**1.80 [1.18, 2.74]**	**2.50 [1.74, 3.59]**	**2.77 [1.49, 5.17]**	**0.93 (0.20)**	**2.51 [1.68, 3.74]**
Sex (0 = men)	**1.78 [1.47, 2.16]**	1.14 [0.93, 1.40]	1.09 [0.87, 1.36]	**3.85 [2.71, 5.46]**	**0.32 (0.06)**	**1.35 [1.12, 1.63]**
Age	**1.01 [1.00, 1.02]**	1.00 [1.00, 1.01]	1.00 [1.00, 1.01]	1.01 [1.00, 1.02]	**0.01 (0.00)**	**0.97 [0.97, 0.98]**
Education level	**0.87 [0.07, 0.20]**	0.94 [0.85, 1.04]	**0.90 [0.81, 0.99]**	0.93 [0.82, 1.06]	−**0.08 (0.03)**	**0.83 [0.74, 0.94]**

*Note.* Bold adjusted odds ratios represent significance for dichotomized experiences with childhood trauma and bullying-victimization (0 = not experienced, 1 = experienced). Bold unstandardized regression coefficients for childhood trauma severity represent significance; a higher score represents more frequent and more types of childhood trauma experiences.

a Childhood trauma severity scores range from 0 to 8. Controlling for gender (0 = men, 1 = women), age, and education level.

**Table 3a:** Lifetime DSM-IV disorders by sexual attraction, controlling for childhood trauma severity (N = 6392).

	Lifetime prevalence of DSM-IV disorders			
	aOR [95%CI]	aOR [95%CI] controlling childhood trauma severity^a^	Indirect effect	% of effect explained
Any mood disorder	**2.64 [1.80, 3.88]**	**2.11 [1.44, 3.08]**	<.001	28.7%
Major depression	**2.17 [1.41, 3.36]**	**1.75 [1.17, 2.60]**	<.001	31.7%
Bipolar disorder	**4.00 [1.06, 15.04]**	2.37 [0.43, 13.24]	.006	22.5%
Dysthymia	**3.66 [1.91, 7.01]**	**2.38 [1.20, 4.74]**	.001	25.5%
Any anxiety disorder	**2.38 [1.49, 3.80]**	**1.90 [1.15, 3.12]**	<.001	29.2%
Social phobia	**2.99 [1.79, 4.99]**	**2.31 [1.27, 4.20]**	<.001	23.7%
Specific phobia	1.51 [0.78, 2.94]			
Panic disorder	1.62 [0.94, 2.79]			
Agoraphobia (without panic)	1.84 [0.54, 6.22]			
Generalized anxiety disorder	2.32 [0.97, 5.55]			
Any substance	**2.08 [1.32, 3.26]**	**1.69 [1.03, 2.80]**	<.001	25.3%
Substance abuse	**1.57 [1.02, 2.44]**	1.29 [0.84, 1.99]	.001	35.7%
Substance dependence	2.53 [0.96, 6.66]			
Any Axis-1 disorder	**1.59 [1.13, 2.24]**	1.27 [0.88, 1.82]	<.001	57.0%
		*B* (*SE*) controlling childhood trauma severity^a^		
Number of Axis-1 disorders	0.58 (0.14)***	0.41 (0.14)**	<.001	31.4%

*Note*. Analyses present comparisons between same/both-sex attraction (1) and exclusively other-sex attraction (0), controlling for gender (0 = men, 1 = women), age, and education level. Differences between predominantly other-sex attraction and exclusively other-sex attraction were non-significant and are presented in Supplemental Table 2.

a Childhood trauma severity was added into the models of DSM-IV disorders for which there was a significant difference by sexual attraction. Bold adjusted odds ratios and regression coefficients represent significance. Percentage explained is calculated using the following formula: $\frac{a\cdot \;b}{ab\;+\;c^{\prime }}$

**Table 3b:** 12-Month DSM-IV disorders by sexual attraction, controlling for childhood trauma severity (N = 6392).

	12-month prevalence of DSM-IV disorders			
	aOR [95%CI]	aOR [95%CI] controlling for childhood trauma severity^a^	Indirect effect	% of effect explained
Any mood disorder	**3.44 [1.92, 6.18]**	**2.55 [1.38, 4.71]**	<.001	21.0%
Major depression	**3.09 [1.63, 5.87]**	**2.33 [1.29, 4.23]**	<.001	20.6%
Bipolar disorder	4.05 [0.72, 22.61]			
Dysthymia	2.51 [0.92, 6.81]			
Any anxiety disorder	**2.63 [1.58, 4.39]**	**2.09 [1.16, 3.76]**	<.001	24.1%
Social phobia	**4.20 [1.85, 9.56]**	**3.21 [1.28, 8.02]**	<.001	14.5%
Specific phobia	0.97 [0.51, 1.84]			
Panic disorder	**2.71 [1.14, 6.42]**	1.87 [0.74, 4.71]	.007	37.7%
Agoraphobia (without panic)	1.82 [0.21, 16.02]			
Generalized anxiety disorder	1.41 [0.64, 3.09]			
Any substance	1.91 [0.85, 4.28]			
Substance abuse	0.95 [0.41, 2.22]			
Substance dependence	**4.68 [1.27, 17.31]**	3.34 [0.66, 16.90]	.017	9.0%
Any Axis-1 disorder	**2.64 [1.77, 3.94]**	**2.12 [1.38, 3.24]**	<.001	24.8%
	*B* (*SE*)	*B (SE)* controlling childhood trauma severity^a^		
Number of Axis-1 disorders	0.32 (0.10)**	0.23 (0.11)*	<.001	28.4%

*Note.* Analyses present comparison between same/both-sex attraction (1) and exclusively other-sex attraction (0), controlling for gender (0 = men, 1 = women), age, and education level. Differences between predominantly other-sex attraction and exclusively other-sex attraction were non-significant and are presented in Supplemental Table 2.

a Childhood trauma severity was added into the models of DSM-IV disorders for which there was a significant difference by sexual attraction. Bold adjusted odds ratios and regression coefficients represent significance. Percentage explained is calculated using the following formula: $\frac{a\;\cdot b}{ab\;+\;c^{\prime }}$

**Table 4:** Lifetime and 12-month DSM-IV disorders by sexual attraction, controlling for bullying-victimization (N = 6392).

	Lifetime prevalence of DSM-IV disorders			12-month prevalence of DSM-IV disorders		
Predictor: Same/both-sex attraction (1), other-sex attraction (0)	aOR [95%CI] controlling for bullying-victimization^a^	Indirect effect	% of effect explained	aOR [95%CI] controlling for bullying-victimization^a^	Indirect effect	% of effect explained
Any mood disorder	**2.33 [1.53, 3.54]**	.001	14.5%	**2.99 [1.59, 5.61]**	.004	10.2%
Major depression	**1.94 [1.24, 3.05]**	.002	15.2%	**2.75 [1.43, 5.31]**	.025	8.5%
Bipolar disorder	2.97 [0.61, 14.44]	.013	14.1%			
Dysthymia	**3.19 [1.68, 6.07]**	.019	9.3%			
Any anxiety disorder	**2.07 [1.26, 3.40]**	<.001	18.5%	**2.29 [1.30, 4.05]**	<.001	15.4%
Social phobia	**2.55 [1.43, 4.56]**	.001	14.7%	**3.61 [1.49, 8.77]**	.001	8.7%
Panic disorder				**2.41 [1.03, 5.67]**	.165	
Any substance	**1.95 [1.23, 3.09]**	.045	6.6%			
Substance abuse	1.52 [0.99, 2.33]	.264				
Substance dependence				**4.30 [1.03, 17.97]**	.313	
Any Axis-1 disorder	1.42 [0.98, 2.04]	.001	26.8%	**2.37 [1.54, 3.64]**	.001	12.4%
	*B* (*SE*) controlling for bullying-victimization^a^			*B* (*SE*) controlling for bullying-victimization^a^		
Number of Axis-1 disorders	0.50 (0.14)**	<.001	15.4%	0.28 (0.11)*	.001	13.7%

*Note.* Controlling for gender (0 = men, 1 = women), age, and education level.

a Bullying-victimization was added into the models of DSM-IV disorders for which there was a significant difference by sexual attraction. Indirect associations are only assessed when differences in DSM-IV disorders by sexual attraction were significant, see Table 3ab. Bold adjusted odds ratios and regression coefficients represent significance. Percentage mediated is calculated using the following formula: $\frac{a\;.b}{ab\;+\;c^{\prime }}$
