# Supplementary material for: Childhood trauma and bullying-victimization as an explanation for differences in mental disorders by sexual orientation
Source: J Psychiatr Res. Author manuscript; Available in PMC 2022 Jun 4. (PMC7612811; doi:10.1016/j.jpsychires.2021.02.046)
Supplement: Appendix A [file EMS145488-supplement-Appendix_A.docx]

Supplemental Table 1

*Weighted Percentages of Lifetime and 12-Month DSM-IV Disorders by Sexual Attraction*

|  | Exclusively other-sex attracted | Predominantly other-sex attracted | Both-sex attracted | Predominantly same-sex attracted | Exclusively same-sex attracted |
| --- | --- | --- | --- | --- | --- |
|  | % | % | % | % | % |
| *Lifetime prevalence of DSM-IV disorders* | | | | | |
| Any mood disorder | 19.30 | 23.14 | 57.44 | 31.64 | 26.56 |
| Major depression | 17.92 | 21.67 | 44.73 | 28.48 | 23.73 |
| Bipolar disorder | 1.23 | 1.47 | 12.72 | 3.16 | 0.73 |
| Dysthymia | 1.22 | 1.80 | 7.86 | 0.00 | 3.27 |
| Any anxiety disorder | 19.12 | 23.45 | 49.57 | 32.36 | 27.43 |
| Social phobia | 8.79 | 10.66 | 29.79 | 19.43 | 18.81 |
| Specific phobia | 7.82 | 8.87 | 18.40 | 16.04 | 4.93 |
| Panic disorder | 3.70 | 4.19 | 4.60 | 10.04 | 5.34 |
| Agoraphobia (without panic) | 0.83 | 2.07 | 4.12 | 0.00 | 0.00 |
| General anxiety disorder | 4.45 | 5.20 | 17.07 | 8.39 | 4.97 |
| Any substance | 19.11 | 13.85 | 41.57 | 21.56 | 26.34 |
| Substance abuse | 16.70 | 11.73 | 26.87 | 15.51 | 22.29 |
| Substance dependence | 3.64 | 2.97 | 15.72 | 6.05 | 4.04 |
| Any Axis-1 disorder | 42.30 | 43.79 | 65.63 | 46.03 | 47.88 |
| *12-month prevalence of DSM-IV disorders* | | | | | |
| Any mood disorder | 5.73 | 7.86 | 33.87 | 13.76 | 7.42 |
| Major depression | 4.87 | 7.25 | 24.05 | 13.76 | 6.69 |
| Bipolar disorder | 0.77 | 0.60 | 9.82 | 0.00 | 0.73 |
| Dysthymia | 0.90 | 1.09 | 5.03 | 0.00 | 1.18 |
| Any anxiety disorder | 9.67 | 13.90 | 28.13 | 18.92 | 19.33 |
| Social phobia | 3.43 | 5.32 | 21.56 | 9.71 | 8.61 |
| Specific phobia | 5.09 | 6.15 | 3.37 | 10.07 | 4.93 |
| Panic disorder | 1.10 | 2.24 | 2.08 | 3.16 | 3.44 |
| Agoraphobia (without panic) | 0.37 | 0.48 | 1.99 | 0.00 | 0.00 |
| General anxiety disorder | 1.72 | 2.31 | 2.21 | 4.02 | 2.35 |
| Any substance | 5.60 | 4.75 | 12.29 | 16.40 | 7.11 |
| Substance abuse | 4.55 | 3.52 | 1.02 | 10.35 | 5.38 |
| Substance dependence | 1.19 | 1.81 | 11.27 | 6.05 | 1.73 |
| Any Axis-1 disorder | 17.31 | 21.74 | 47.19 | 31.23 | 29.07 |

Supplemental Table 2

*Lifetime and 12-Month DSM-IV Disorders For Predominantly Other-Sex Attracted and Exclusively Other-Sex Attracted Individuals*

|  | Lifetime prevalence of DSM-IV disorders | 12-month prevalence of DSM-IV disorders |
| --- | --- | --- |
| Predominantly other-sex attraction versus exclusively other-sex attraction | aOR [95%CI] | aOR [95%CI] |
| Any mood disorder | 1.09 [0.80, 1.47] | 1.24 [0.77, 1.42] |
| Major depression | 1.09 [0.80, 1.48] | 1.36 [0.83, 2.24] |
| Bipolar disorder | 1.14 [0.47, 2.75] | 0.71 [0.20, 2.46] |
| Dysthymia | 1.21 [0.52, 2.78] | 0.98 [0.34, 2.84] |
| Any anxiety disorder | 1.17 [0.89, 1.55] | 1.35 [0.88, 2.08] |
| Social phobia | 1.14 [0.76, 1.73] | 1.46 [0.74, 2.90] |
| Specific phobia | 1.01 [0.67, 1.50] | 1.08 [0.65, 1.78] |
| Panic disorder | 1.01 [0.61, 1.68] | 1.90 [0.88, 4.11] |
| Agoraphobia (without panic) | 2.04 [0.93, 4.49] | 1.02 [0.24, 4.29] |
| General anxiety disorder | 1.08 [0.64, 1.82] | 1.22 [0.55, 2.67] |
| Any substance | 0.88 [0.59, 1.30] | 1.01 [0.63, 1.59] |
| Substance abuse | 0.86 [0.57, 1.28] | 0.94 [0.51, 1.73] |
| Substance dependence | 1.05 [0.58, 1.89] | 1.74 [0.90, 3.36] |
| Any Axis-1 disorder | 1.07 [0.83, 1.37] | 1.30 [0.90, 1.88] |
|  | *B* (*SE*) | *B* (*SE*) |
| Number of Axis-1 disorders | 0.03 (0.06) | 0.06 (0.05) |

*Note*. Controlling for gender (0 = men, 1 = women), age, and education level. Analyses present comparisons between predominantly other-sex attraction versus exclusively other-sex attraction, from models in Tables 3ab.
